# Two Sides to the Same Coin—Cytotoxicity vs. Potential Metastatic Activity of AgNPs Relative to Triple-Negative Human Breast Cancer MDA-MB-436 Cells

**DOI:** 10.3390/molecules25102375

**Published:** 2020-05-20

**Authors:** Magdalena Matysiak-Kucharek, Magdalena Czajka, Barbara Jodłowska-Jędrych, Krzysztof Sawicki, Paulina Wojtyła-Buciora, Marcin Kruszewski, Lucyna Kapka-Skrzypczak

**Affiliations:** 1 Department of Molecular Biology and Translational Research, Institute of Rural Health, 20-090 Lublin, Poland; czajka.magdalena@imw.lublin.pl (M.C.); sawicki.krzysztof@imw.lublin.pl (K.S.); lucynakapka@gmail.com (L.K.-S.); 2 Department of Histology and Embryology with Experimental Cytology Unit, Medical University of Lublin, 20-059 Lublin, Poland; b.jedrych@gmail.com; 3 The President Stanisław Wojciechowski State University of Applied Sciences, 62-800 Kalisz, Poland; paulinawojtyla@gmail.com; 4 Center for Radiobiology and Biological Dosimetry, Institute of Nuclear Chemistry and Technology, 03-195 Warsaw, Poland; marcin.kruszewski@gmail.com

**Keywords:** MDA-MB-436, breast cancer, silver nanoparticles, cytotoxicity, oxidative stress, inflammation, actin cytoskeleton, metastasis

## Abstract

Silver nanoparticles (AgNPs) are used in many fields of industry and medicine. Despite the well-established antimicrobial activity, AgNPs are foreseen to be used as anticancer drugs due to the unusual feature—inability to induce drug resistance in cancer cells. The aim of the study was to assess biological activity of AgNPs against MDA-MB-436 cells. The cells were derived from triple-negative breast cancer, a type of breast cancer with poor prognosis and is particularly difficult to cure. AgNPs were toxic to MDA-MB-436 cells and the probable mechanism of toxicity was the induction of oxidative stress. These promising effects, giving the opportunity to use AgNPs as an anti-cancer agent should, however, be treated with caution in the light of further results. Namely, the treatment of MDA-MB-436 cells with AgNPs was associated with the increased secretion of several cytokines and chemokines, which were important in breast cancer metastasis. Finally, changes in the actin cytoskeleton of MDA-MB-436 cells under the influence of AgNPs treatment were also observed.

## 1. Introduction

According to the World Health Organization, breast cancer is the most commonly diagnosed female cancer in the world and the most common cause of cancer-related deaths among women [1]. An extremely fatal subtype of breast cancer is a triple-negative breast cancer (TNBC), characterized by a lack of expression of the estrogen receptor (ER), progesterone receptor (PR) and non-elevated expression of the human epidermal growth factor receptor (HER2). This diagnosis is associated with a poor prognosis and lack of effective targeted therapy, as well as a high tendency to create distant metastasis [2]. Due to limited therapeutic options, new forms of treatment are constantly being sought. In recent years, the attention of oncologists and scientists has focused on nanotechnology. Nanoparticles (NPs) are used in diagnostics and serve as carriers of cytostatic drugs, or amplifiers for radio- and photodynamic therapy [3,4,5]. NPs also show anti-tumor activity per se, demonstrating cyto- and genotoxic effects and inducing apoptosis in tumor cells [6,7].

Due to their unique physicochemical properties, NPs are widely used not only in oncology but in many fields of science and industry. One of the most well-known and widely used NPs are silver nanoparticles (AgNPs), which have excellent antibacterial, antiviral and antifungal properties, and are successfully used in many commercial products [8]. Therefore, the general population is exposed to AgNPs released into the environment [9]. AgNPs enter the human body mainly by inhalation and ingestion. Once in the body, AgNPs accumulate in various internal organs, especially the liver, spleen, lungs, kidneys and brain [10,11].

The general toxicity of AgNPs was tested in various in vitro models, both in cells isolated from normal tissues or cancer-derived lines [12,13,14]. Also, in vivo AgNPs presented a wide spectrum of toxic effects [15,16]. There are no human population studies that would help to assess the risks associated with exposure to AgNP and other NPs. In vitro studies on AgNPs toxicity focus mainly on the assessment of cell viability and proliferation, induction of apoptosis, ROS production and DNA damage. Nevertheless, it should be remembered that NPs, apart from the possible cytotoxic and/or genotoxic results, may also induce other unintended effects. This may, for example, be the induction of an inflammatory process leading to the secretion of different cytokines, chemokines and acute-phase proteins. An acute immune response, resulting from the presence of a pathogen, is beneficial and its task is to eliminate the harmful agent. However, chronic inflammation triggers cellular events which can promote carcinogenesis and tumor progression. Increasingly, cancer is treated as an inflammatory process that proceeds in a dynamic microenvironment, contributing to metastasis [17,18]. The NPs impact on the activity of cellular signaling pathways is also poorly understood [19]. Recently, some reports indicate that NPs can stimulate metastasis by modulation of epithelial-mesenchymal transition (EMT) [20]. During EMT, the cell cytoskeleton undergoes a dynamic remodeling, the expression of many proteins changes and the cell increases its ability to move and migrate. EMT can be initiated by various agents, e.g., pro-inflammatory cytokines and growth factors [21].

Considering the above reports, the aim of our study was to assess the biological activity of AgNPs against MDA-MB-436 triple-negative metastatic human breast cancer cells. We examined the effect of AgNPs on the viability of MDA-MB-436 cells and the level of oxidative stress and apoptosis biomarkers. Next, we measured the effect of AgNPs on the secretion of cytokines, chemokines and acute-phase proteins. Finally, we investigated AgNPs impact on the actin cytoskeleton of MDA-MB-436 cells.

## 2. Results

### 2.1. AgNPs Cytotoxicity

**Figure 1:**
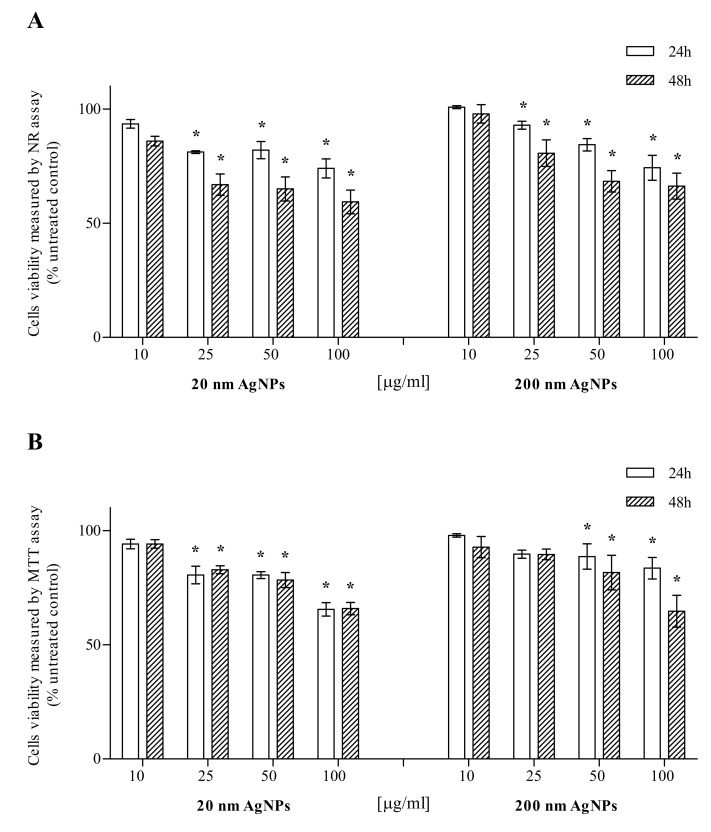
Cytotoxic effect of 20 or 200 nm AgNPs on the MDA-MB-436 cell line. Viability was measured using NR (**A**) and MTT (**B**) assays and presented as a percentage of the untreated control. Data were expressed as mean ± standard deviation (*n* = 3). Statistical significance: *p < 0.05.

MDA-MB-436 cells were treated with 20 nm or 200 nm AgNPs in the concentration range of 10–100 μg/mL, while AgNPs untreated cells (0 μg/mL) served as a control for viability studies. Cell viability was evaluated after 24 and 48 h incubation by the NR and MTT assays. The NR assay showed that 20 and 200 nm AgNPs are toxic to MDA-MB-436 cells, depending on the time of incubation and the NPs concentration. Statistical analysis showed a significant decrease in the viability of MDA-MB-436 cells after treatment with 20 or 200 nm AgNPs at concentrations of 25–100 μg/mL (Figure 1A). The MTT assay also showed that 20 or 200 nm AgNPs were toxic to MDA-MB-436 cells in the incubation time and NPs concentration-dependent manner. Statistical analysis revealed a significant viability decrease after 25–100 µg/mL incubation with 20 nm AgNPs, both after 24 and 48 h. The effect of 200 nm AgNPs was significant in 50 and 100 µg/mL (Figure 1B).

### 2.2. Oxidative Stress Markers

**Figure 2:**
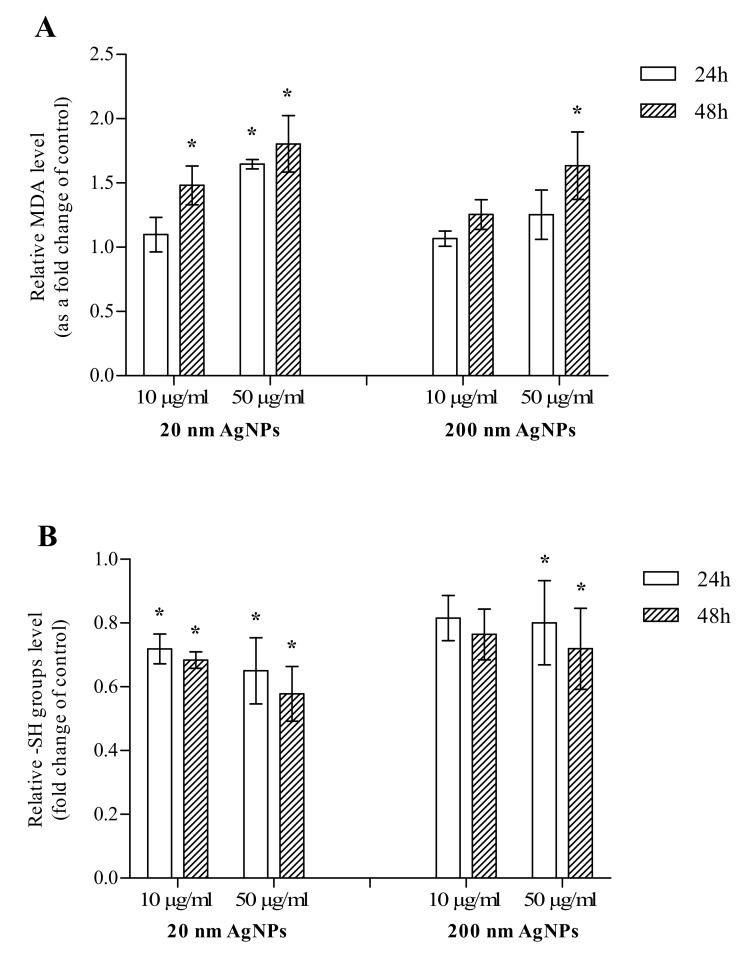
The level of malondialdehyde (MDA) (**A**) and thiols (-SH groups) (**B**) in MDA-MB-436 cells treated with 20 or 200 nm AgNPs. The graph presents the fold change of MDA and -SH groups level calculated for samples incubated with AgNPs relative to untreated control. The data were expressed as mean ± standard deviation (*n* = 3). Statistical significance: * *p* < 0.05.

The impact of 20 and 200 nm AgNPs on the formation of MDA and thiols levels in MDA-MB-436 cells was measured after 24 and 48 h incubation. After a 24 h incubation, a statistically significant increase in MDA was observed only when cells were incubated with 50 μg/mL 20 nm AgNPs, however, an upward trend was also observed for 50 μg/mL 200 nm AgNPs. After a 48 h incubation, an increase in the MDA level was statistically significant in all concentrations and sizes tested, except for 10 μg/mL 200 nm AgNPs (Figure 2A). After 24 and 48 h incubations, a statistically significant decrease in the thiol (-SH groups) level was observed in MDA-MB-436 cells treated with 10 or 50 μg/mL 20 nm AgNPs or 50 μg/mL 200 nm AgNPs. For 10 μg/mL 200 nm AgNPs, the decrease was also observed; however, it did not reach statistical significance (Figure 2B). In summary, 20 and 200 nm AgNPs influenced both investigated oxidative stress markers, while the effect of smaller NPs was more pronounced in both cases.

### 2.3. Apoptosis

**Figure 3:**
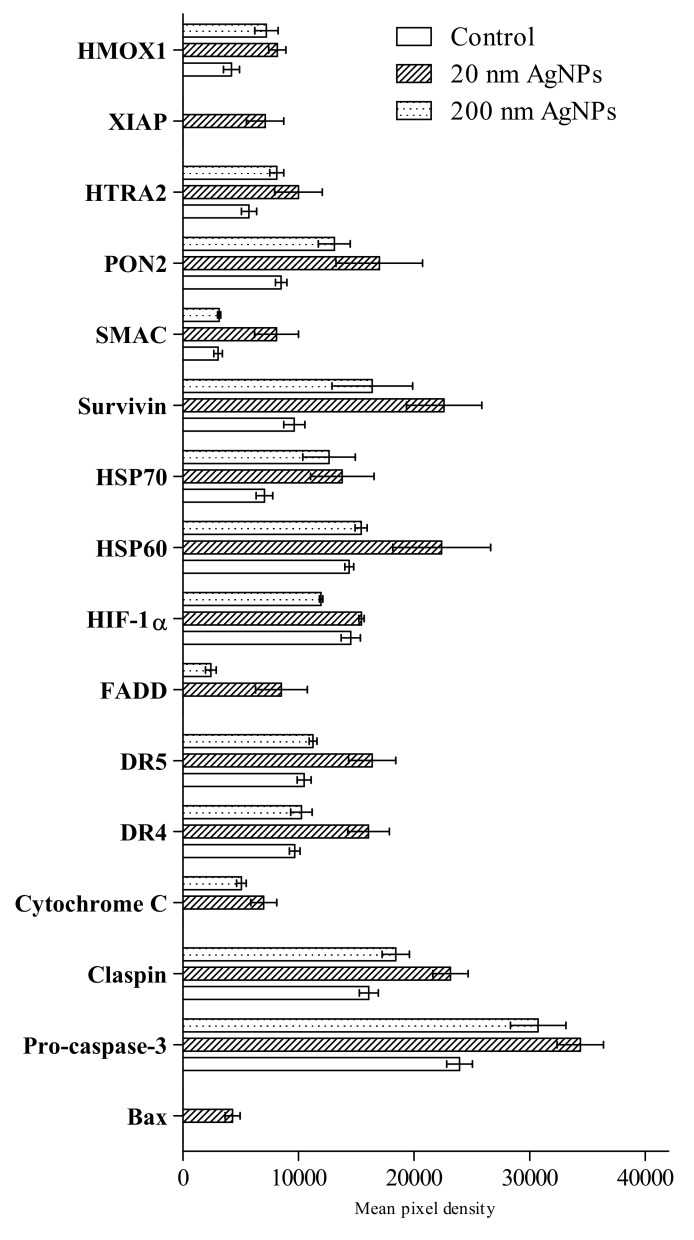
Semi-quantitative assessment of apoptosis markers in MDA-MB-436 cells after incubation with 20 or 200 nm AgNPs measured by the Proteome Profiler Human Apoptosis Array Kit in a mixture of cells lysates from three independent experiments. Untreated cells were used as a control. Apoptosis markers levels were presented as the mean with the range of two individual measurements, normalized to reference spots and the negative control (film background) was subtracted.

An analysis using Proteome Profiler Human Apoptosis Array Kit revealed the presence of 16 out of 35 tested proteins involved in apoptosis (Figure 3). AgNPs treatment affected heme oxygenase 1 (HMOX1), paraoxonase 2 (PON2), secondary mitochondria-derived activator of caspases (SMAC), survivin, heat shock protein 60 (HSP60), heat shock protein 70 (HSP70), hypoxia-inducible factor 1-alpha (HIF-1a), death receptor 5 (DR5), death receptor 4 (DR4), cytochrome C, claspin and pro-caspase-3. Protein expression of X-linked inhibitor of apoptosis (XIAP), Fas-associated protein with death domain (FADD), cytochrome C and BCL2-associated X protein (Bax) were not detected in untreated cells, however, those factors were detected in cells after AgNPs stimulation.

### 2.4. Inflammation

**Figure 4:**
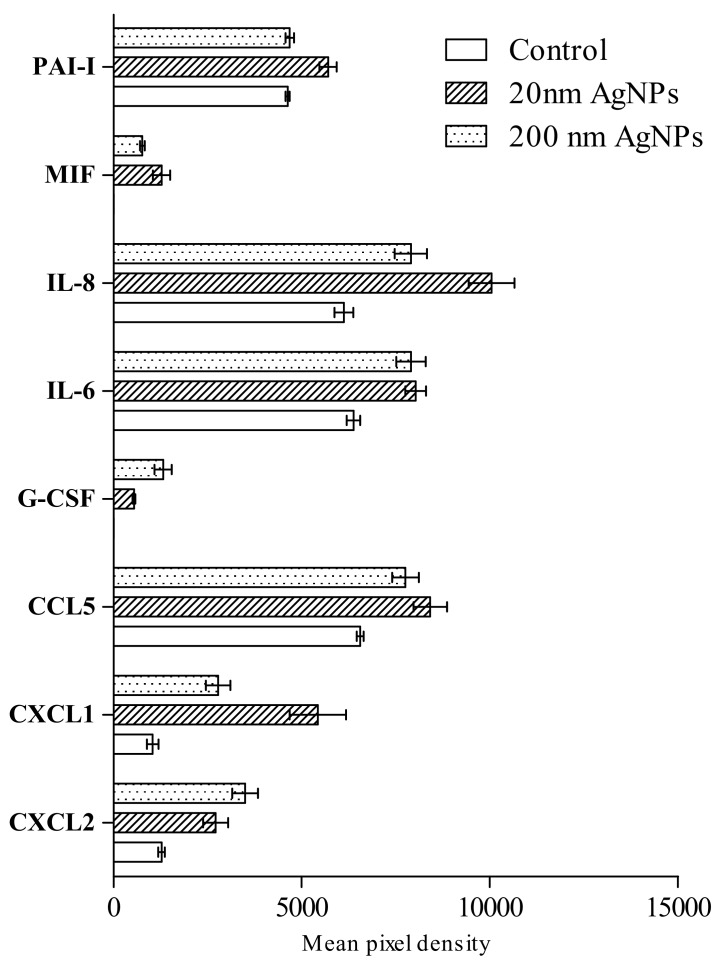
Semi-quantitative assessment of inflammatory markers secreted by MDA-MB-436 after incubation with 20 or 200 nm AgNPs measured by Human Profiler Cytokine Array Kit in a mixture of cells media from three independent experiments. Untreated cells were used as a control. Cytokine levels were presented as the mean with the range of two individual measurements, normalized to reference spots, and the negative control (film background) was subtracted.

An analysis using the Human Profiler Cytokine Array Kit revealed the presence of 8 of 36 tested proteins that are involved in the inflammatory process (Figure 4). The treatment with AgNPs increased secretion of CC motif chemokine ligand 2 (CCL2), chemokine (CC motif) ligand 1 (CXCL1), chemokine (CC motif) ligand 5 (CXCL5), interleukin 6 (IL-6), interleukin 8 (IL-8) and plasminogen activator inhibitor-1 (PAI-1). Secretion of granulocyte colony stimulating factor (G-CSF) and macrophage migration inhibitory factor (MIF) was not detected in untreated cells, however, a low level of both factors was detected in the medium after stimulation. Secretion of CXCL1 and IL-8 was more intense after stimulation with 20 nm AgNPs than 200 nm, in other cases the effect was similar.

**Figure 5:**
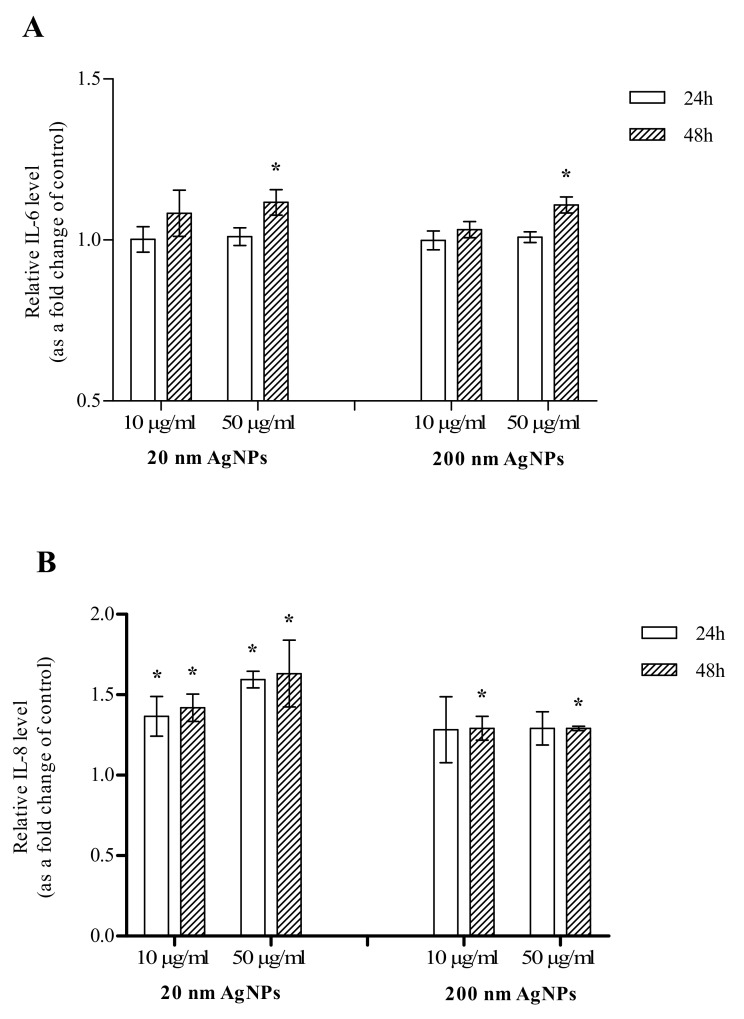
Interleukin 6 (IL-6) (**A**) and interleukin 8 (IL-8) (**B**) levels in growth media of MDA-MB-436 cells treated with 20 or 200 nm AgNPs. The graph presents the fold change of IL-6/IL-8 level calculated for samples incubated with AgNPs, relative to untreated control. Data were expressed as mean ± standard deviation (*n* = 3). Statistical significance: * *p* < 0.05.

The effect of AgNPs on the secretion of IL-6 was additionally confirmed by the ELISA assay. There was no change in IL-6 levels in AgNPs-treated cells after 24 h incubation. After 48 h incubation of MDA-MB-436 cells with 20 or 200 nm AgNPs, an increase in IL-6 secreted by the cells was observed. Although in cells incubated with both diameters of 50 μg/mL AgNPs the increase reached statistical significance, the observed effect was very slight (Figure 5A). The effect of AgNPs on the secretion of IL-8 was also confirmed by the ELISA assay. After a 24 h incubation, there was a statistically significant change in 20 nm AgNPs-treated cells both at 10 and 50 µg/mL concentrations. The increase was also observed for 200 nm AgNPs but did not reach statistical significance. After a 48 h incubation of MDA-MB-436 cells with 20 or 200 nm AgNPs, the increase in IL-8 secreted by the cells reached statistical significance in all tested concentrations (Figure 5B).

### 2.5. Impact of AgNPs on Cytoskeleton Organization

**Figure 6:**
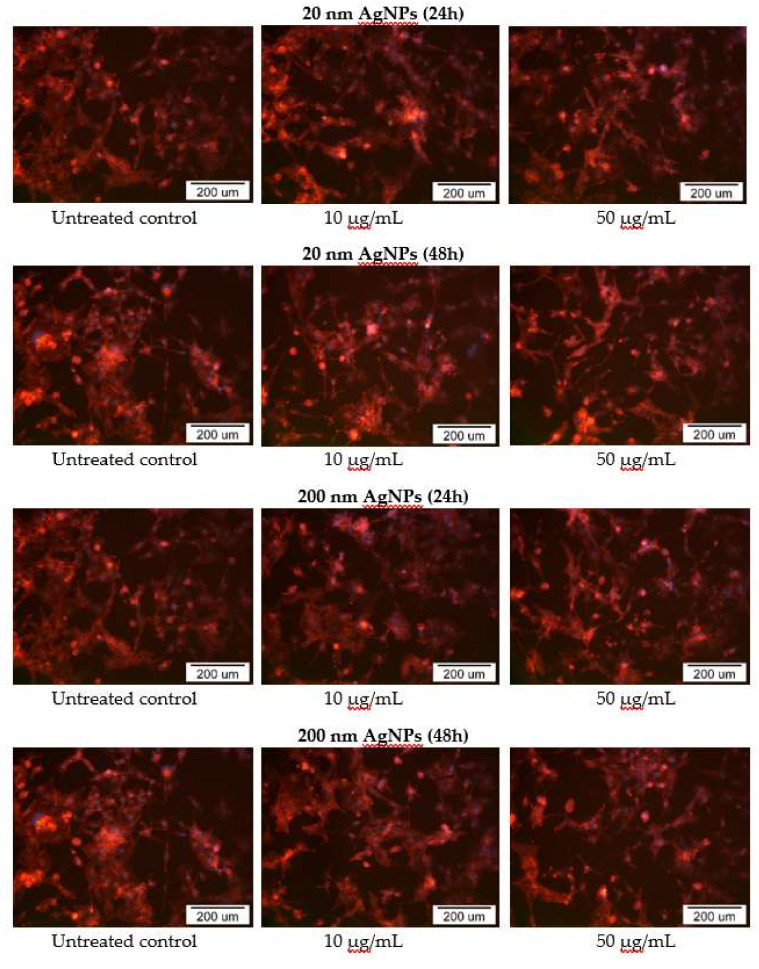
Phalloidin-TRICT staining of MDA-MB-436 cells actin cytoskeleton after incubation with 20 and 200 nm AgNPs solutions. Additional DAPI staining was performed to dye cells nuclei.

The impact of 20 and 200 nm AgNPs on MDA-MB-436 actin cytoskeleton was also examined using fluorescent microscopy. In AgNPs treated cells, disruption of the actin cytoskeleton was observed in part of cells, which correlates with previously demonstrated cytotoxicity. In addition, changes in cell morphology, such as a decrease in cell adhesion, presence of cells with an elongated shape and the formation of actin branches at the ends of cells, were also noted, especially in cells treated with high (50 μg/mL) concentrations of AgNPs after a 48 h incubation (Figure 6).

## 3. Discussion

There are numerous reports indicating that AgNPs affect cell viability. The AgNPs cytotoxicity depends on numerous factors e.g., type of cells and growth media used in the studies, AgNPs size, shape, functionalization, coating, exposure time, concentration, solubility, distribution of particles and aggregation state [22,23]. Therefore, the AgNPs cytotoxic activity should be assessed individually for each experimental model. In our research, we showed that 20 and 200 nm AgNPs significantly affect the viability of MDA-MB-436 cells in a dose and time-dependent manner. The results were confirmed both in the NR assay, investigating the integrity of cell membranes (Figure 1A) and the MTT test, assessing the activity of mitochondrial cell metabolism (Figure 1B). In our studies, two AgNPs dimensions—20 and 200 nm were compared, as size is a critical NP parameter. In general, a number of studies showed that AgNPs toxicity is increased with decreased diameters. Considering the same mass, smaller NPs have a larger specific surface area, where interactions with cellular components may occur. The smaller size also makes it easier for the nanoparticle to enter the cell [24].

The cytotoxicity of AgNPs is primarily associated with the induction of oxidative stress. It has been demonstrated in numerous cellular models that treatment with AgNPs leads to overproduction of reactive oxygen species (ROS) and to inactivation of the antioxidant defense system, which is manifested, for example, by a decrease in the concentration of reduced glutathione [12,25]. Induction of oxidative stress leads to the damaging of various cellular components, lipid membrane peroxidation, protein carbonylation or DNA breaks, which can lead to necrotic cell death. It was also found that the oxidative stress induced by AgNPs is an important factor directing cells to the pathway of apoptosis [24,26]. In our hands, the incubation of MDA-MB-436 cells with AgNPs also led to a statistically significant elevated level of malondialdehyde, which is the final lipid peroxidation product (Figure 2A). In addition, we showed that AgNPs treatment led to a statistically significant decrease in the number of thiol groups, the most important representative of which is reduced glutathione (Figure 2B). This result, in turn, shows that AgNPs interfere with the antioxidant defense mechanisms in MDA-MB-436 cells. It has also been shown that in AgNPs treated MDA-MB-436 cells the level of a number of proapoptotic proteins (HIF-1α, SMAC, HTRA2, DR4, DR5, BAX, Cytochrome C, FADD and TNF-RI) is increased in relation to cells grown in medium without NPs. At the same time, an increase in several anti-apoptotic proteins (HSP60, HSP70, PON2, HXMO1, XIAP, Survivin and Claspin) expression was demonstrated. Therefore, it is possible that cells treated with AgNPs developed some anti-apoptotic mechanisms, which has also been observed in other tumor cell lines as a result of prolonged AgNP exposure [27].

To the best of our knowledge, this is the first time that the cytotoxic effect of AgNPs on MDA-MB-436 cells has been demonstrated. We also did not find any studies that investigated the oxidative stress parameters or apoptosis markers in this experimental model. Other NPs were also not tested in MDA-MB-436 cells but showed cytotoxicity in other TNBC cells [28]. Returning to AgNPs, Swanner et al. [29] demonstrated that AgNPs coated with polyvinylpyrrolidone (PVP) decrease the viability of TNBC cells—MDA-MB-231, BT549 and SUM-159. The MTT assay, confirmed by the BrdU and flow cytometry, were used to determine cytotoxicity. For MDA-MB-231 cells, which showed the highest susceptibility, the level of oxidized thiol groups was also measured. In PVP-AgNPs treated MDA-MB-231 cells, their level was increased which is indicative of oxidative stress. Also, Juarez-Moreno et al. [30] revealed the cytotoxic effect of PVP-AgNPs on MDA-MB-231 cells. In addition, they showed that PVP-AgNPs caused an increase in the amount of ROS, apoptosis and induction of DNA damage. As mentioned earlier, AgNPs toxicity studies are a complex topic and it is difficult to compare them, especially considering the coating of NPs. AgNPs can be coated with various inorganic and organic substances. The coating provides greater stability and prevents aggregation of NPs due to electrostatic interactions. It has been proven that various types of coatings significantly affect the AgNPs cytotoxicity, mainly reducing it compared to uncoated particles [31]. It seems, therefore, that AgNPs are capable of inducing cytotoxicity, oxidative stress and apoptosis in TBNC cells. However, TNBC cell lines may present a significantly different molecular profile. Both the MDA-MB-436 and MDA-MB-231 lines belong to the molecular subtype of claudin-low breast cancer characterized by low expression of markers associated with cell–cell interaction such as claudine 3, 4 and 7, occludin or E- cadherin, high expression of mesenchymal EMT markers and stem cell traits. However, the MDA-MB-436 cells are less aggressive, and the cells are not tumorigenic in immunosuppressed mice [28].

Since the induction of the inflammatory process may be directly related to ROS production, we investigated the effect of AgNPs on the secretion of cytokines, chemokines and acute phase proteins. AgNPs increased the secretion of several proteins that had a significant role in the process of tumor metastasis (Figure 4). The high level of IL-8 secretion is associated with the invasiveness and aggressiveness of breast cancer cells in vitro and in vivo [32,33], their overexpression can also lead to EMT induction [34]. Another chemokine, of which secretion was elevated by AgNPs treatment, was CXCL-1 that promotes breast cancer migration and its invasive capacity, as well as EMT in human breast cancer cells and in vivo animal models [35]. In addition, secretion of CCL2 protein was detected, which is over-expressed in invasive breast cancer and regulates its progression [36]. Furthermore, the secretion of IL-6 and CCL5 was slightly increased in AgNPs treated MDA-MB-436 cells. The important role of those two proteins was described in TNBC tumor growth and metastasis, suggesting that both promote crosstalk between TNBC cells and lymphatic vessels [37]. Moreover, IL-6 is closely associated with the induction of EMT [38]. In addition, AgNPs treated MDA-MB-436 cells secreted G-CSF and MIF. Both proteins have the potential to promote metastasis of breast cancer cells [39,40].

In terms of secreted cytokines, the impact of AgNPs on MDA-MB-436 cells mimics the effects of tumor-associated macrophages (TAMs). TAMs produce multiple growth factors and inflammatory cytokines (IL–1β, IL–6 and TNF–α), each associated with EMT in cancer cells. On the other hand, the CXCL1 and IL–8 gradient produced by AgNPs treated MDA-MB-436 cells may attract myeloid-derived suppressor cells (MDSCs) that preferentially infiltrate the primary tumor site than metastases. The MDSCs could promote EMT-like changes in cancer cells, via multiple pathways [41]. Finally, secretion of IL-6 and IL-8 by AgNP treated cells stimulates its own EMT via a feedback loop type interaction. IL–8 binds to CXCR1/2 receptors and activates several signaling pathways, e.g., Ras/Raf/MAPK, PI3K, or JAK/STAT that promote EMT. IL–8-mediated EMT has been demonstrated in breast cancer [42]. The potential of IL–6 of inducing and maintaining EMT in breast cancer cell lines depends on the activation of the JAK2/STAT3 pathway or tropomyosin receptor kinase C (TrkC) that triggers EMT and activates Twist1-mediated IL–6 overexpression, securing the EMT phenotype via a feedback loop interaction [38,43]. So far, no other studies have been conducted regarding the effect of AgNPs and other NPs on the level of the above-described proteins, not only in MDA-MB-436 cells, but also other models of breast cancer. However, there are numerous reports on the pro-inflammatory [44] and anti-inflammatory effects of AgNPs in various in vitro models [45]. The anti-inflammatory effect of AgNPs is mainly related to NPs obtained by green synthesis from various types of plant extracts. Apart from the fact that studies on the NPs effects depend on many factors and must be considered individually, properties of AgNPs obtained by green synthesis may be conditioned not only by AgNP action but also by specific compounds contained in plant extracts. Therefore, the obtained results are encouraging, but there is a need for consecutive, more detailed research. Primarily, the level of all cytokines and chemokines should be quantified in a wider concentration range, as performed for IL-6 (Figure 5A) and IL-8 (Figure 5B).

In addition to cytotoxicity, oxidative stress parameters, apoptosis and pro-inflammatory markers, the actin cytoskeleton morphology was examined in AgNPs-treated MDA-MB-436 cells. It should be noted that MDA-MB-436 cells have a mixed phenotype, with both epithelial and mesenchymal characteristics. The reorganization of the cytoskeleton observed in MDA-MB-436 cells can potentially lead to a change in cell phenotype to mesenchymal, which may increase their ability to move and migrate (Figure 6). Whereas no data seems to be available for AgNPs so far, similar results were demonstrated for carbon nanotubes, silicon oxide and titanium oxide NPs in various in vitro models [46]. In the MDA-MB-231 cells treatment with fullerenol resulted in the decreased number and length of filopodia [47]. In the same experimental model treated with gadolinium metallofullerenol, the cells changed their phenotype towards epithelial [48]. Although these results are opposite to ours, it should be noted that fullerenols are specific carbon-based NPs, characterized by low toxicity and anti-oxidant properties [49].

In conclusion, we have demonstrated for the first time the impact of AgNPs on human triple-negative breast cancer MDA-MB-436 cells. We have also shown that AgNPs are cytotoxic to cancer cells and the potential mechanism of their toxicity is a disruption of the oxidative balance. Moreover, AgNP-treated MDA-MB-436 cells enter the path of apoptosis. These effects raise hopes for the use of AgNPs as an anti-cancer agent, however, they should be treated cautiously, since we have also shown that AgNP-treated MDA-MB-436 cells developed some anti-apoptotic changes and secrete increased amounts of several pro-inflammatory proteins associated with breast cancer metastasis. AgNPs also led to changes in cells morphology, which may indicate an increased metastatic potential. Therefore, it seems that although the use of nanomaterials in the diagnosis and treatment of cancer is a great hope of modern medicine, we must not forget that the risk associated with exposure to NPs is still insufficiently known, as demonstrated in this paper for AgNPs.

## 4. Materials and Methodology

### 4.1. Materials

#### 4.1.1. Nanoparticles Preparation

In our studies, commercially available, non-coated AgNPs of nominal size 20 or 200 nm (both PlasmaChem GmbH, Berlin, Germany) were used. NPs stock solutions (2 mg/cm^3^) were prepared by dispersion of 2 mg of AgNPs in 800 µL of distilled water. Particles dispersions were sonicated on ice using a Vibra-Cell 130 probe sonicator (Sonics & Materials Inc, Newton, CT, USA). Then, 100 µL of 10x concentrated phosphate-buffered saline and 100 µL of 15% bovine serum albumin solution was added, according to our previous studies [50]. Intermediate dilutions were made by diluting the stock solution in an appropriate cell medium. Detailed characteristics of the used AgNPs in different culture media can be found in previous papers. This method of preparation used was to ensure the highest possible stability of NPs as AgNPs tend to aggregate, especially smaller ones quickly form larger complexes and their real dimensions in the cellular medium are not homogeneous but are in the range of sizes [50,51].

#### 4.1.2. Cell Culture

The studies were conducted on MDA-MB-436 cells—TNBC derived, pleural effusion origin, infiltrating ducal carcinoma and of claudin-low molecular subtype. The cell line was purchased from Cell Lines Service (CLS, Hamburg, Germany) and cells were grown according to the manufacturer′s instructions in a humidified atmosphere of 95% air and 5% CO_2_ at 37 °C. DMEM: Ham F-12 in a 1:1 ratio supplemented with L-glutamine, penicillin-streptomycin, and 10% fetal bovine serum was used as growth medium (all from Sigma Aldrich, Saint Louis, MO, USA).

### 4.2. Cytotoxicity Studies

#### 4.2.1. Neutral Red Assay

MDA-MB-436 cells were seeded in 96-well microplates (TPP, Trasadingen, Switzerland) at a density of 2 × 10^4^ cells/well in 200 μl of cell culture medium. The cells were incubated overnight, the medium was then replaced with freshly prepared AgNPs solutions. After a suitable time of treatment (24 or 48 h), the medium was aspired, cells were rinsed with 150 µl of PBS and incubated with 100 µl of neutral red (NR) solution (50 μg/mL) for 4 h at 37 °C. Subsequently, the NR solution was removed, cells were washed with 150 µL of PBS and 200 µL of an acetic acid ethanol solution (50% ethanol, 49% water and 1% acetic acid) was added to each well. After 20 min of gentle shaking, NR absorbance was measured spectrophotometrically at 540 nm using the Omega FLUOstar microplate reader (BMG LABTECH, Ortenberg, Germany).

#### 4.2.2. MTT Assay

MDA-MB-436 cells were seeded in 96-well microplates (TPP, Trasadingen, Switzerland) at a density of 1 × 10^4^ cells/well in 100 μl of culture medium. At 24 h after cell seeding, cells were treated with suitable AgNPs solutions. After 24 or 48 h incubation, 15 μL of 3-(4,5-dimethylthiazol-2-yl)-2,5-diphenyltetrazolium bromide (MTT) of 5 mg/mL stock solution was added to each well and incubated for 3 h at 37 °C. The fluid was then removed and 100 µL of dimethyl sulfoxide (DMSO) was added to each well. After 2 min of intense shaking, optical density was read at 570 nm by the Omega FLUOstar microplate reader (BMG LABTECH, Ortenberg, Germany).

### 4.3. Oxidative Stress Markers

The appropriate numbers of MDA-MB-436 cells were seeded on 6-well cell culture plates and left overnight to obtain optimal attachment to the surface. Subsequently, fresh AgNPs stock suspension (or medium without NPs for control plates) was added (10 or 50 μg/mL AgNPs). After 24 or 48 h, medium was aspired, cells were washed with PBS and trypsinized, collected and transferred into Eppendorf tubes. Then, cells were centrifuged (12,000× *g* for 10 min at 4 °C), the supernatant was discarded and the pellet was washed twice with PBS, then precipitated cells were frozen at −80 °C for further analysis. Cells lysates were prepared by suspending cells pellets in radioimmunoprecipitation assay buffer (RIPA) (Thermo Fisher Scientific Inc., Waltham, MA, USA) supplemented with a protease and phosphatase inhibitors cocktail.

#### 4.3.1. Malondialdehyde

Malondialdehyde (MDA) level in lysates of MDA-MB-436 cells treated with 20 and 200 nm AgNPs was determined using a commercially available, based on reaction with thiobarbituric acid, QuantiChromTM TBARS Assay Kit (BioAssay Systems, Hayward, CA, USA), as recommended by the manufacturer. Cells cultured in medium without the addition of NPs were the control. The spectrophotometric analysis of the reaction product at 535 nm was carried out using an Omega FLUOstar microplate reader (BMG LABTECH, Ortenberg, Germany).

#### 4.3.2. Thiol Compounds

The level of thiol compounds (-SH groups) was determined in lysates of MDA-MB-436 cells treated with 20 and 200 nm AgNPs, using the Fluorometric Thiol Assay Kit (Sigma-Aldrich, Saint Louis, MO, USA). The analysis, based on the Elman′s reaction, was carried out in accordance with the manufacturer’s instructions. Cells cultured in medium without the addition of NPs were the control. The fluorimetric analysis of the reaction product was carried out using an Omega FLUOstar microplate reader (BMG LABTECH, Ortenberg, Germany).

### 4.4. Apoptosis

The semi-quantitative Proteome Profiler Human Apoptosis Array Kit (R&D Systems, Minneapolis, MN, USA) assay was used for the determination of the apoptosis markers in MDA-MB-436 cells treated with 50 μg/mL 20 or 200 nm AgNPs for 48 h. AgNPs-treated MDA-MB-436 cells lysates from three independent experiments were mixed together, then used in the Proteome Profiler Human Apoptosis Array Kit, according to the manufacturer’s protocol. In the next step, membranes containing apoptosis-related antibodies were developed on the Carestream^®^ Bio Max^®^ light film (Kodak, Rochester, NY, USA) and the intensity of specific protein points was quantified using commercially available Image J software (National Institutes of Health, Bethesda, MD, USA). The result for protein was presented as the average of two corresponding points, after subtracting the negative control (background intensity) and normalizing the result to the positive control (reference points).

### 4.5. Inflammation

MDA-MB-436 cells were seeded on 6-well cell culture plates and left overnight to obtain optimal attachment to the surface. Subsequently, AgNPs stock suspension (or medium without nanoparticles for control plates) was added (10 or 50 μg/mL AgNPs). After incubation for 24 or 48 h, the cell culture medium was collected and centrifuged (12,000× *g* for 10 min at 4 °C) to remove NPs, and frozen at −80 °C for subsequent enzyme-linked immunosorbent (ELISA) and Human Profiler Cytokine Array Kit assays.

#### 4.5.1. Human Profiler Cytokine Array Kit

For the determination of the protein profile in culture media of MDA-MB-436 cells treated with 50 μg/mL of 20 or 200 nm AgNPs for 48 h, the commercially available Human Profiler Cytokine Array Kit (R&D Systems, Minneapolis, MN, USA) was used according to the manufacturer′s instructions. Since this is a semi-quantitative assay and three independent replicates were not performed, in order to obtain the most consistent average result, the conditioned media from the MDA-MB-436 cells treated with AgNPs in three independent experiments were mixed together, and this mixture was used in the Human Profiler Cytokine Array Kit assay. For visualization, membranes were developed on the Carestream^®^ BioMax^®^ light film (Kodak, Rochester, NY, USA). Then, the intensity of specific protein points was quantified using commercially available Image J software (National Institutes of Health, Bethesda, MD, USA). The average of two points corresponding to one protein was calculated, background intensity was subtracted (negative control) and protein intensity was normalized to reference points (positive control).

#### 4.5.2. IL-6 and IL-8

IL-6 and IL-8 release was analyzed in the culture media of MDA-MB-436 cells exposed to 20 or 200 nm AgNPs for 24 or 48 h using Human IL-6 ELISA Kit (Sigma-Aldrich, Saint Louis, MO, USA) and Human IL-8 ELISA Kit (Abcam, Cambridge, UK) following the manufacturer′s protocol. Spectrophotometric analysis of the reaction product was carried out at 450 nm using an Omega FLUOstar microplate reader (BMG LABTECH, Ortenberg, Germany).

### 4.6. Actin Cytoskeleton Staining

To evaluate 20 and 200 nm AgNPs impact on the actin cytoskeleton of the MDA-MB-436 cells, the cells were grown on 8-well cell culture slides (Nunc^®^ Lab-Tek^®^ Chamber Slide™, Thermo Fisher Scientific Inc., Waltham, MA, USA) overnight, then treated with appropriate AgNPs solutions. After 24 or 48 h incubation, cells were fixed with 3.7% paraformaldehyde and permeabilized with 0.1% Triton X. The presence of actin filaments was evaluated using phalloidin-tetramethylrhodamine B isothiocyanate (Sigma Aldrich, Saint Louis, MO, USA). The cell nuclei were additionally stained with DAPI (Sigma Aldrich, Saint Louis, MO, USA). Fluorescence labeling was analyzed under a fluorescent microscope Olympus BX51 (Olympus Corporation, Tokyo, Japan).

### 4.7. Statistical Analysis

With the exception of the results of Proteome Profiler Human Cytokine Array and Proteome Profiler Human Apoptosis Array, the obtained results were subjected to statistical analysis using the GraphPad Prism 5.0 software (GraphPad Software Inc., San Diego, CA, USA). The data were expressed as mean ± standard deviation. At least three independent experiments were conducted. Statistical significance was evaluated using a one-way analysis of variance (ANOVA). Then, if necessary, Tukey′s test was used for a post-hoc comparison. Differences were defined as statistically significant when the *p*-value was <0.05. All graphs were also prepared in GraphPad Prism 5.0 software (GraphPad Software Inc., San Diego, CA, USA).
